# The combination of high Q factor and chirality in twin cavities and microcavity chain

**DOI:** 10.1038/srep06493

**Published:** 2014-09-29

**Authors:** Qinghai Song, Nan Zhang, Huilin Zhai, Shuai Liu, Zhiyuan Gu, Kaiyang Wang, Shang Sun, Zhiwei Chen, Meng Li, Shumin Xiao

**Affiliations:** 1 Department of Electrical and Information Engineering, Harbin Institute of Technology, Shenzhen, China; 2 State Key Laboratory on Tunable Laser Technology, Harbin Institute of Technology, Harbin, China; 3 Department of Material Science and Engineering, Harbin Institute of Technology, Shenzhen, China

## Abstract

Chirality in microcavities has recently shown its bright future in optical sensing and microsized coherent light sources. The key parameters for such applications are the high quality (Q) factor and large chirality. However, the previous reported chiral resonances are either low Q modes or require very special cavity designs. Here we demonstrate a novel, robust, and general mechanism to obtain the chirality in circular cavity. By placing a circular cavity and a spiral cavity in proximity, we show that ultra-high Q factor, large chirality, and unidirectional output can be obtained simultaneously. The highest Q factors of the non-orthogonal mode pairs are almost the same as the ones in circular cavity. And the co-propagating directions of the non-orthogonal mode pairs can be reversed by tuning the mode coupling. This new mechanism for the combination of high Q factor and large chirality is found to be very robust to cavity size, refractive index, and the shape deformation, showing very nice fabrication tolerance. And it can be further extended to microcavity chain and microcavity plane. We believe that our research will shed light on the practical applications of chirality and microcavities.

Optical microcavities have attracted considerable research attention due to their potential applications in micro-lasers and solid-state cavity quantum electrodynamics^1^. Whispering gallery (WG) modes based microcavities are particular interesting because of their ultra-high Q factors and relative small mode volumes. The record high Q factors are on the order of 10^6^ for semiconductor microdisk^2^, 10^8^ for microtoroid^3^, and 10^9^ for microsphere^4^. In WG cavities, light is trapped along the cavity boundary for a long time by total internal reflection. Due to the presence of chiral symmetry, the propagating waves along clockwise (CW) and counter-clockwise (CCW) directions have equal optical lengths and contribute equally to the field distributions of hybrid modes^5^,^6^. Except for several special designs such as limaçon cavity^7^,^8^,^9^, the balance between CW and CCW components usually gives multiple directional emissions, even though the cavity shapes have been strongly deformed to quadruple^10^, stadium^11^,^12^, and oval shapes^13^,^14^.

Spiral shaped microcavity is a special type of deformed cavity^15^,^16^. Due to the lack of any discrete spatial symmetry, the modes in spiral cavity have been demonstrated to be highly non-orthogonal pairs and each pair show strong spatial chirality^15^,^16^,^17^. In 2003, Chern et al have utilized the chirality to generate unidirectional laser emission^15^. Soon after, people realized that the chirality also happened in the quasi-scarred modes^17^. And the almost-degenerate mode pairs with mainly CCW characters have been experimentally observed^18^. In past few years, the chirality has been observed in deformed cavities without mirror-reflection symmetries^19^, microdisk perturbed by external scatters^20^,^21^, and parity-time-symmetric quantum rings^22^. And the asymmetrical scattering efficiencies between CW and CCW components have been found to play crucial roles in forming the chirality^17^. Very recently, the applications of chirality have also been successfully extended from micro-lasers^15^,^23^ to label-free optical detectors^24^ and fundamental researches on exceptional points (EP)^25^,^26^. However, the current designs of microcavities with chirality face severe challenges from practical applications. The Q factors of spiral cavity are usually low because of the strong scattering at the notch^15^. The deformed limaçon and microdisk perturbed by external nanoparticles can support high-Q chiral resonances. But only a few of them can have large chirality, for instance, α > 0.8^19^,^20^. Till now, there is a lack of a general mechanism to obtain ultra-high Q factor and large chirality from multi-modes simultaneously.

Here we demonstrate a novel, robust, and general mechanism to obtain the chirality in optical microcavities. By placing a circular cavity and a spiral cavity in proximity, our numerical calculations show that ultra-high Q factor, chirality, and unidirectional output can be obtained simultaneously. The highest Q factors of the non-orthogonal mode pairs are almost the same as the orthogonal ones in a pure circular cavity. And the co-propagating directions of the non-orthogonal mode pairs can be reversed by counting the mode coupling in photonic molecule. At last, we show that the new mechanism can be further extended to microcavity chain and microcavity plane.

## Results and Discussion

In additional to a single cavity, twin disks have also been intensely studied in past decade^27^,^28^,^29^,^30^,^31^,^32^,^33^. In analogy to the homonuclear diatomic molecule such as H_2_, the interaction between two identical cavities has been experimentally studied in 1998^27^,^28^. Symmetric and anti-symmetric resonances have been observed and the possibility in spectral engineering has triggered a number of practical applications such as optical sensing and optical gyroscope^29^,^30^. Soon after, the heteronuclear diatomic photonic molecule has also been proposed by coupling two size mismatched cavities^31^,^32^,^33^,^34^. Besides the spectral engineering, the size mismatched photonic molecule has also served as a nice platform to study fundamental concepts such avoided resonance crossing and exceptional point^31^,^32^,^33^,^34^. Most importantly, each cavity can contribute different properties to the hybrid mode, giving more degrees to tailor the resonant behaviors within optical microcavity e.g. the high Q factor and the large chirality.

### Chirality in photonic molecule

**Figure 1:**
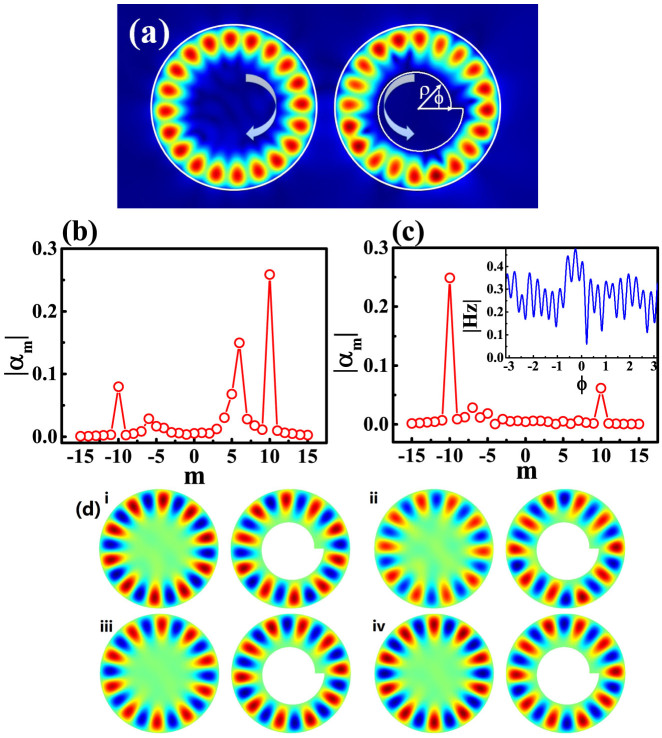
(a) the schematic picture of photonic molecule. The radiuses of left and right circles are R1 and R2, respectively. The boundary of spiral shape is defined as 
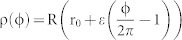
, here r_0_ = 0.56, R2 = R, R1 = 0.9985R, d = 0.2R, and ε = 0.16. The separation distance between two cavities is d. (b) and (c) show the normalized angular momentum distribution |α_m_| of resonance at kR ~ 4.3436 − 0.001086i in annular ring (b) and circular cavity (c), respectively. (d) The field distribution (Hz) of four hybrid modes within the photonic molecules. The arrows in (a) indicate the main propagating directions. The inset in (c) shows the distribution of |Hz| along the boundary of left circle. Note that ε = 0.16 is not particularly selected. Similar phenomena have also been observed in other shape deformations such as 0.12 ≤ ε ≤ 0.17.

The first structure of the twin disks is depicted as the white lines in Fig. 1(a). It consists of a circular disk with radius R1 and an annular ring. The outer boundary of annular ring is R2 and the inner boundary is a spiral, which is defined as 
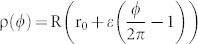
 in polar coordinates. The light within the annular ring will be scattered by the notch of the inner boundary. As the thicknesses of microdisk are usually much smaller than their diameter, the three-dimensional structures of microdisks can be simplified to two-dimensional shapes by using effective refractive index n. In the following, we set n = 3.3 [for GaAs] for the circular disk and annular ring. The other places are air with n = 1. It is easy to find that the scattering from CW waves to CCW waves at the notch is much larger than the reversed process^15^. Then the chirality and non-orthogonality can be easily expected due to the presence of asymmetrical scattering efficiencies. Meanwhile, as the light is well confined by total internal reflection in the circular cavity, the Q factors of modes in circular cavity can be extremely high as conventional WG modes. Therefore, it is interesting to explore the possibility to combine the high Q factor and larger chirality in such photonic molecule.

We then numerically calculate the resonant frequencies (ω) of TE (transverse electric) modes in photonic molecule by solving the Helmholtz equation^35^. Similar to the typical proximity resonance^36^, the degeneracy of WG modes in circular cavity is removed when the circular cavity is brought close to annular ring. Four non-degenerate hybrid modes can still be formed by the mode coupling^31^. But their field distributions are different from previous reports about twin disk. Taking the modes at kR ~ 4.3436 as an example [see Fig. 1(d)], we can still see the symmetric and anti-symmetric field distributions within two cavities. But their symmetries with respect to axis along ϕ = 0 disappear. The vanishment of mirror-reflection symmetry of the modes in annular ring is consistent with its structure and the corresponding chirality^15^,^17^. It is more interesting to see that the mirror-reflection symmetry of field distributions are also absent in the circular ring, indicating the dissemination of the chirality from annular ring to circular cavity. Similar inkling has also been found in the field distribution (|Hz|) of in Fig. 1(a). We can see that the minima values between two nodes don't reach to zero [see the exact value in the inset in Fig. 1(c)]. As the standing waves of WG modes are generated by the interference between CW and CCW components, t he non-zero |Hz| value clearly shows the imbalance between the CW and CCW propagating waves.

To confirm the chirality in circular disk, we analyzed the field distribution (Hz) by expanding the wave functions inside the cavity in cylindrical harmonics^15^,^38^, 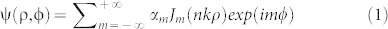
where *J_m_* is the *m*th-order Bessel function of the first kind. Positive and negative values of angular momentum *m* correspond to the CCW and CW propagating components, respectively. Figure 1(b) shows the angular momentum distribution |α_m_| of the resonance in Fig. 1(a). We can see that the resonance is dominated by the CCW components with *m = 10*, confirming the chirality in annular ring. Meanwhile, the angular momentum distribution in circular cavity [Fig. 1(c)] also shows strong chirality. By define the chirality as 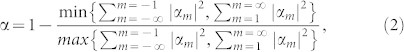
we find that the chiralitites of the field distributions in annular ring and circular cavity are both around 0.9. Therefore, the co-propagating directions of non-orthogonal modes pair can be schematically depicted by the gray arrows in Fig. 1(a). The waves of the hybrid modes mainly propagate along CW direction in circular cavity and along CCW direction in the annular ring. And the chirality is transferred by the proximity resonance.

**Figure 2:**
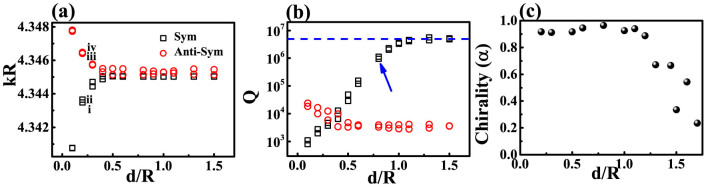
The resonant frequencies (a), Q factors (b), and chirality of long-lived mode (c) as a function of separation distance d. All the other parameters are the same as Fig. 1. The solid line in (b) is the Q factor of the same resonance within circular cavity only.

As the proximity resonance is usually sensitive to the separation distance^36^, we then studied the resonant behaviors of the hybrid modes in twin disks. All the calculated results are presented in Fig. 2. With the increasing of separation distance from 0 to 0.4R, the resonant frequencies of symmetric and anti-symmetric modes shift to higher and lower energy, respectively. Meanwhile, the Q factors of symmetric and anti-symmetric modes increase and decrease. All the changes are consistent with typical behavior of mode coupling^36^. The phases of anti-symmetric modes within two cavities have π difference. Then the destructive interference will cancel the field distribution within the gap area. Thus more fields will be pushed back into the cavities and give higher frequencies and Q factors.

**Figure 3:**
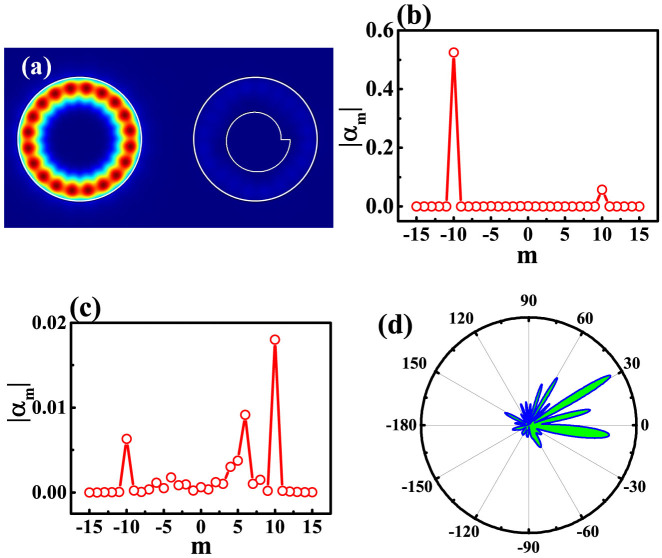
The field pattern (a), angular momentum distribution in circle (b), and angular momentum distribution in annular ring (c) of in twin disks. All the parameters are the same as Fig. 1 except for d = 0.8R. While the angular momentum within annular ring is similar to Fig. 1, the main distribution in circular cavity is quite different. The component with |m| = 6 reduces to 0. (d) The corresponding far field pattern of the same resonance.

While the mode coupling is quite dramatic when 0 < d < 0.4R, the maxima Q value is only around 10^4^, which is far below the requirements for optical sensing^24^ and our expectations. This is also caused by the strong scattering loss at the notch^15^. Moreover, for the lasing mode at 930 nm, the d = 0.4R corresponds to a separation distance only around 250 nm, which is not easy for standard photolithography. We then focus on the range d > 0.4R. In this range, the resonant frequencies of symmetric and anti-symmetric are very close and the mode coupling turns to be negligible. The corresponding field patterns [see Fig. 3(a) as an example] also show that their fields are primarily confined within circular cavity or annular ring. As the resonances confined in circular cavity are less influenced by the annular ring, their Q factors [black squares in Fig. 2(b)] gradually increase and approach to the values of conventional WG modes in a single circular cavity and keep as a constant when d > 1.0. Interestingly, the chirality is almost kept as a constant value within a wide range 0.1 < d < 1.2. It doesn't vanish when the mode coupling is very weak. Therefore, it is possible to achieve the ultrahigh Q factor and large chirality simultaneously in a wide range 0.8 < d < 1.2. And the dissemination of chirality can be beyond of proximity resonance.

We then studied the resonant properties of modes at d = 0.8R to understand the formation of chirality in circular cavity at this region. The spatial field distribution |Hz| is plotted in Fig. 3(a). While most of energy is confined well within the circular cavity, the fluctuation of |Hz| is very weak and shows strong difference with conventional WG modes. Figure 3(b) confirms the difference by expanding the wavefunctions. We can see that most of energy locates at m = −10 and the calculated chirality is as high as 0.99. While the mode coupling is weak in this region, such a large chirality is still not very surprising. As the separation distance is less than a wavelength, the evanescent waves can reach the annular ring and circulate inside it. As depicted in Fig. 3(c), the annular momentum distributions of evanescent waves follow the resonance within the annular ring and give a similar chirality to Fig. 1(b). Compared with the direct tunneling, the influence of coupled evanescent waves in annular ring is much stronger and thus can dominate the chiral properties of circular cavity. This can be further confirmed by the unidirectional output in Fig. 3(d), which is completely formed by the scattering at the notch^15^.

### Control the co-propagating direction of non-orthogonal mode pair

**Figure 4:**
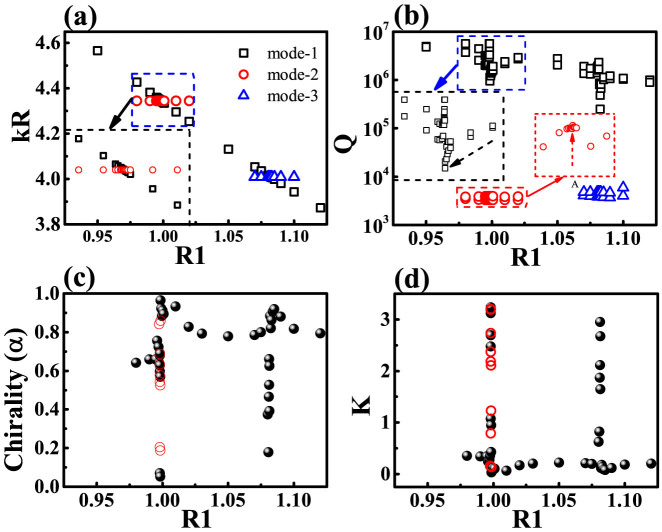
The dependences of resonant frequencies (a), Q factors (b), K factors (c), and chiralities (d) on the size of circular cavity. At R1 ~ 0.99813 and R1 ~ 1.0812, the co-propagating directions of non-orthogonal mode pairs dramatically transit to a reversed direction. The open circles in (c) and (d) are the chirality and 1/K of evanescent waves inside the annular ring. Insets in (a) and (b) show the enlarged figures of frequency crossing and linewidth repulsion. The reduction and increase of Q factors of mode-1 and mode-2 are marked by two dashed arrows in the insets of Fig. 4(b).

Similar to the studies of size-mismatched photonic molecule, the coupling between circular cavity and annular ring can also be used to explore some fundamental phenomena such as avoided resonance crossing and exceptional point^14^,^17^,^24^,^25^,^26^. In general, avoided resonance crossing has two scenarios. The strong coupling situation exhibits a frequency repulsion and a linewidth crossing. The weak coupling situation consists of a frequency crossing and a linewidth repulsion. Keeping all the other parameters the same as Fig. 3 and changing R1, we have also observed such kind of behaviors. As shown in Figs. 4(a) and 4(b), the WG modes in circular cavity gradually approach two resonances [marked as mode-2 and mode-3] in annular ring and cross with them. Meanwhile, we also notice that the Q factors of mode-1 and mode-2 have clear reductions and slight increases around the crossing points (see the enlarged figures in the insets of Fig. 4(b)), clearly demonstrating the repulsion in linewidths (Q ∝ 1/Δν, where Δν is the linewidth). We thus know that weak coupling happens between the circular cavity and the annular ring. Tuning the separation distance and refractive index, it is also possible to find strong coupling and exceptional point, which are similar to previous reports and won't be presented here.

**Figure 5:**
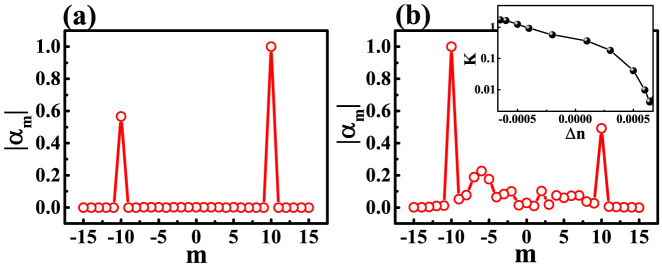
The angular momentum distribution |α_m_| within the circular cavity (a) and annular ring (b) at R1 = 0.99813. Inset in (b) is the K factor as a function of Δn. With a slight change Δn ~ 0.0013, the resonance is dramatically switched from CCW dominated components to CW dominated propagating waves.

The interesting phenomenon happens in the chirality. Due to the presence of notch^15^,^16^,^17^, the chiral states in circular cavity mainly along CW direction. This has been confirmed by Figs. 1–3 and most of resonances in Fig. 4. In Fig. 4(c), it is surprising to see that the chirality suddenly reduces to zero around the crossing points. By defining a K parameter as 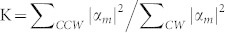, we found that the K factor dramatically increased from K ~ 0 to K > 3 in Fig. 4(d). Similar change holds true for another nearly degenerate mode. This means that the main propagating directions of the non-orthogonal mode pairs have been switched from CW direction to CCW direction. Such changes can be clearly seen from the angular momentum distribution in Fig. 5(a). Different from Fig. 3(b), the components at m = 10 turn to be dominant.

As the circular cavity itself doesn't possess asymmetrical scattering, it is natural to explore the influences of annular ring again. Similar to Fig. 3, the evanescent waves go into the annular ring and experience the asymmetrical scattering at the notch. Figure 5(b) shows the calculated angular momentum distribution |α_m_| of the evanescent waves inside the annular ring. Here we can see that the evanescent wave is dominated by the CW component with m < 0. As the evanescent waves are scattered asymmetrically, the chirality of resonances within circular cavity becomes reasonable. The influence of annular ring has been further confirmed by changing R1. As depicted in Figs. 4(c) and 4(d) [open circles], the chirality and propagating directions are consistent with the changes in circular cavity very well. Most importantly, the change of propagating direction is also very dramatic around the crossing points. In Fig. 4(d), the full widths at half maximum of two narrow peaks are as small as 0.0001R. As the chirality has the potential to be applied as sensors to rotation and nanoparticle^24^, such a dramatic change in propagating direction around the mode coupling can further improve the sensitivity.

Then the key question turns to be the formation of chirality along CW direction inside the annular ring. Different from the circular cavity such as Fig. 5(a), the angular momentum distribution in Fig. 5(b) has two main components locating at *m = −10* and *m = −6*. This means that the waves within annular ring are mainly confined along the conventional WG orbits with *|m| = 10* and *|m| = 6*. As two orbits also have spatial overlap, then the interference between the scattered waves along two orbits must be considered. Our calculations show that the phase difference between the components at *m = −10* and *m = −6* is around *0.61π*, where the phase difference between the components at *m = 10* and *m = 6* is around *1.1π*. We thus know that most of the scattered light along CCW will be destructively cancelled. Then the scattering from CCW waves to CW waves is larger than the reversed process and the chirality along CW direction is finally formed.

**Figure 6:**
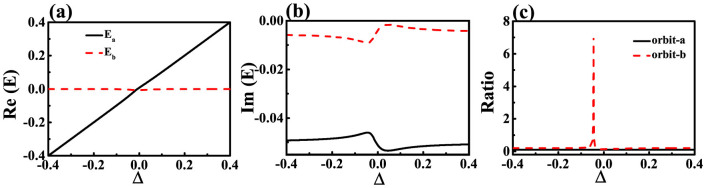
Real (a), imaginary (b) parts of energy E_1CW_, E_2CW_ as a function of energy difference Δ. Here we set Ea = −0.05i, Eb = D − 0.005i, ηa = 0.1, ηb = 0.2, and W1*W2 = −3.3*10^−4^i. (c) shows the ratio between the energy along CCW direction and CW direction of two orbits.

The influence of the interference can be understood with a simple model. For a single orbit, the chirality can be by a 2 × 2 non-Hermitian and non-symmetric matrix^37^

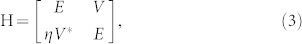
where E is the energy of states in the absence of coupling, V = |*V*|*e^iβ^* describes the scattering from CW to CCW, and ηV* is the scattering rate in the reversed way. Then the corresponding eigenvectors in the CCW and CW traveling-wave basis are 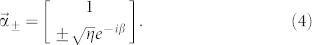
It is thus easy to know that the weight of states rotating in CCW direction and CW direction are 1 and η, respectively. Hence, the chirality in optical cavity is 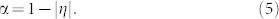
For the case of considering the interference between the waves along two orbits, the model can be rewritten as 4 × 4 matrix 
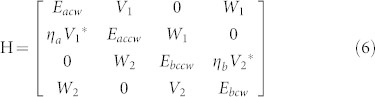
where η_i_ and V_i_ are defined as 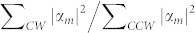 and scattering efficiencies, respectively. E_a_ and E_b_ represent the waves along orbit *a* and orbit *b*. For simplicity, the coupling constants between CW waves (E_acw_ and E_bcw_) and CCW (E_accw_ and E_bccw_) waves are set as the same (W_1_W_2_), and the interaction between E_aCW_ and E_bCCW_ (or E_aCCW_ and E_bCW_) waves are absent. Taking the parameters in Fig. 6 as an example, in additional to the weak coupling in Figs. 6(a) and 6(b), we can also see that the ratio (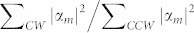) increases from the default value (0.2) to above 6 around the crossing point, indicating the switching from CCW propagation to CW propagation. Moreover, similar to the numerical calculation in Fig. 4(d), such a transition is also very dramatic and happens in a narrow parameter region. Thus the model confirms that the interference between the scattered waves has the possibility to switch the propagating directions of chiral resonances.

Based on above researches, we then can control the chirality of circular cavity via changing local refractive index such as Ref. [35, 38]. Here we applied a perturbation Δn on the circular cavity and studied the responses of propagating waves. The cavity shape is very similar to Fig. 3 except R1 = 0.9983R. All the results are summarized in the inset in Fig. 5(b). We can see that the changing Δn from −0.00065 to 0.00065, the main propagating direction of resonance changes from CCW to CW direction. At Δn = 0.00065, the CW components are almost 250 times larger than the CCW ones. As the slight refractive change can be easily obtained by free carrier injection, thermo optics, or nonlinear effect, changing the local refractive index is useful to fix the possible fabrication deviations and control the propagating direction of chiral resonances.

### Chirality in twin disks

**Figure 7:**
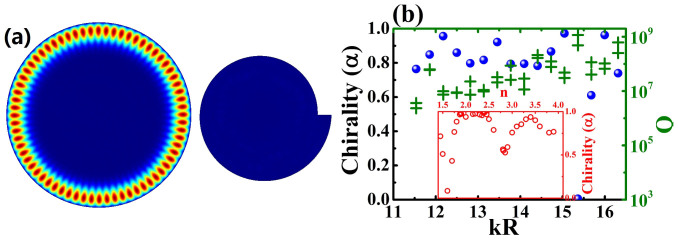
(a) the chirality in conventional twin disk. The radius of circle is R and the boundary of spiral disk is defined with the same equation as Fig. 1. Here r_0_ = 0.75 and ε = 0.15. The separation distance between two cavity is d = 0.1R. (b) The chirality (blue dots) and Q factors (olive crosses) of resonances within the circular cavity. Inset in (b) shows the dependence of the chirality of mode in (a) on the change of refractive index.

Besides the reverse of the co-propagating directions of non-orthogonal mode pairs, Fig. 4(c) confirms one essential information again. Except very narrow regions around the crossing points in Fig. 4(c), the chirality can be observed in a wide range of R1 without any mode coupling. This means the coupling between the circular cavity and annular ring is not a necessary condition for the formation of chirality in circular cavity. Without the coupling to chiral resonance and experience the long time circulating, the asymmetrical scattering of evanescent waves at the notch also has the ability to influence the chirality. Then it is natural to consider a more general case to generate large chirality and high Q factor simultaneously. We present one of such new design in Fig. 7(a), where a circular cavity with radius R and a spiral cavity are placed in proximity. The separation distance between two cavities is *d*. As depicted in Fig. 7(a), the high Q modes of twin disks are mainly confined in the circular cavity and their chiralities have been significantly changed by the asymmetrical scattering of evanescent waves. By expanding the field distribution as Eq. (1), we know its chirality is as high as 0.86 and the main propagation follows CCW direction in circular cavity.

It is interesting to show that the chirality in fig. 7(a) is not unique. Similar chirality can be found in multi-modes. We have calculated the chirality of 16 modes in a range 11 < kR < 17 and summarized the results in Fig. 7(b). Their azimuthal numbers are 23–38 and the radial number is 1. Except the mode at kR ~ 15.3625, all the other modes show quite large chirality. The olive crosses in Fig. 7(b) are the corresponding Q factors. Unlike the oscillation of chirality, the Q factors exponentially increase with kR. This is induced by the different mechanisms for high Q factor and large chirality. Most importantly, all the Q factors in Fig. 7(b) are in a range 10^6^–10^9^. Thus we can conclude that the combination of ultrahigh Q factor and large chirality in such twin disks is quite general.

More than the robustness to the resonant frequency, we have also studied the dependence of chirality on the refractive index. All the results are shown in the inset in Fig. 7(b). We can see that the chirality has been formed in a wide range of refractive index 1.45 < n < 4. Such a wide range means that the material dispersion can be neglected in practical applications. And the twin disks can be fabricated with most of transparent materials including silica, polymer, and the semiconductors. We note that the reduction at n = 1.6 is just caused by mode coupling. It quickly disappears when the resonant frequency changes.

### Chirality in microdisk chain

**Figure 8:**
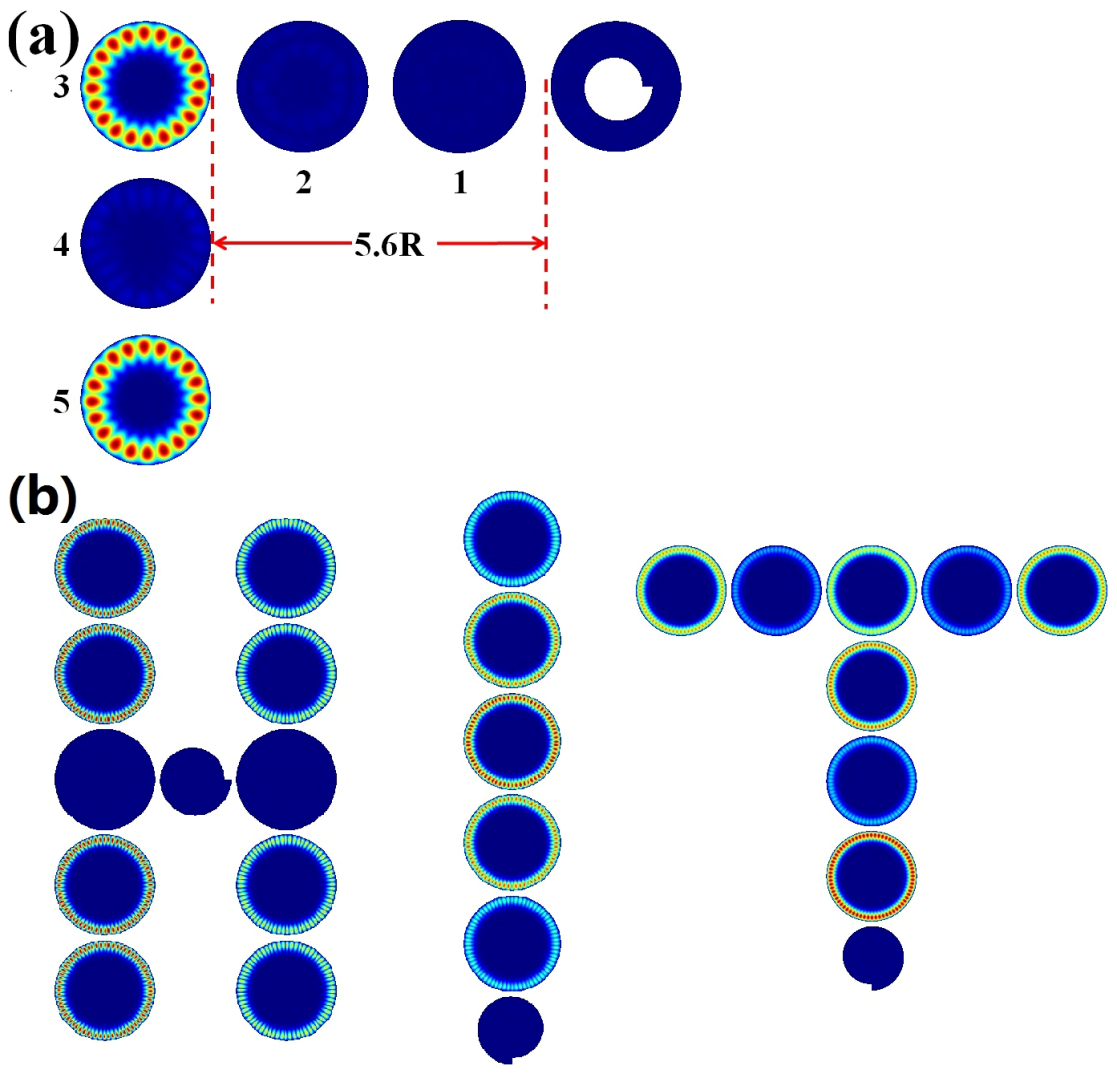
(a) The chirality in microdisk chain with annular ring. The separation distance d = 0.4R and the annular ring is the same as Fig. 1. Here the sizes of disks 1, 2 are 1.02R and 1.01R. The sizes of disks 3–5 are R. (b) The chirality in microdisk chain with the “H”, “I”, “T” shapes. Here the asymmetrical scattering is generated with a spiral cavity. All the parameters are the same as Fig. 7.

More than the simple twin-disk photonic molecule, the chirality can be further extended to multiple cavities. This means that the chirality can be disseminated in a long distance. Figure 8(a) shows the dissemination of chirality from an annular ring to a chain of circular cavities. While the separation distance between disk 3 and annular ring is several times of the resonant wavelength [5.6R], the chirality can still be formed inside the disks 3–5 via the asymmetrical scattering at the notch of annular ring. Meanwhile, as disks 1 and 2 have different sizes with disks 3–5, we thus know that the scattering of evanescent waves can also be transported over a long distance without mode coupling. More than the annular ring, Fig. 8(b) also shows the formation of chirality with spiral cavity. Here we set the sizes of circular cavities and separation distance at R and 0.1R. We can see that the chirality can also be formed in microdisk chains with different shapes such as “H”, “I”, and “T”. As all the sizes of circular cavities are the same, it is possible to excite the chiral resonances within most disks simultaneously. Therefore, placing “HIT” or other shapes together can even push the chiral chain to a chiral plane.

## Conclusion

In summary, we have demonstrated a novel, robust, and general mechanism to obtain the chirality in optical microcavities. Inspired by the so-called heteronuclear diatomic molecule, we design new structures with circular cavities and spiral cavities. The light confinements in former ones usually give ultrahigh Q factors and the asymmetrical scattering of evanescent waves in latter ones generate the chirality. Our calculations show that the ultrahigh Q factors and large chirality can be obtained simultaneously in a wide range of separation distance, cavity size, refractive index, and cavity shape. Such robustness makes the new design easily fabricated in practical applications. Moreover, the mechanism of forming high Q chiral resonances can be further extended to microdisk chain and even microdisk plane.

By introducing mode coupling into the chirality, we have also shown that the co-propagating directions of non-orthogonal mode pairs can be simply switched between CW and CCW directions. As the mode coupling can be easily controlled by changes of local refractive index^35^,^38^, the reverse of the propagating direction can be fulfilled by locally perturbing the cavity. Due to the dramatic change in K factor, the reverse of propagating direction is possible to be utilized to further improve the sensitivity of optical sensors. We believe that our researches will be important for both the practical applications and the fundamental studies around the exceptional points.

## Methods

As the thicknesses of microdisks are much smaller than their in-plane dimensions, microdisks are usually treated as two-dimensional objects by applying effective refractive indices *n*. Then the wave equations for transverse electric (TE, E is in plane) polarized modes *H_z_*(*x, y, t*) = *ψ*(*x, y*)*e^−iωt^* can be replaced by the scalar wave equation 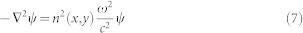
with angular frequency *ω* and speed of light in vacuum *c*. We numerically computed the TE polarized resonances by solving above equation with the RF module in COMSOL Myultiphysics 3.5a. The cavity shape is defined with AutoCAD and imported to the software.

The Q factor of resonance is determined by *Q* = *Re*(*ω*)*/*2*|Im*(*ω*)*|*. And the far field patterns are obtained by calculating the outgoing power flow at the position 20R away from the cavity. Following the expression in Eq. (1) in main text, the angular momentum distribution |α_m_| is obtained by expanding the wavefunctions inside cavity via Fourier transform.

## Author Contributions

Q.S. and S.X. designed the research. Q.S., N.Z., H.Z., S.L., Z.G., K.W., S.S., Z.C., M.L. and S.X. performed the numerical calculation and analysis. Q.S. and S.X. wrote the manuscript and all authors reviewed the contents.
